# Determinants of willingness to undergo aesthetic surgery among Saudi patients: A cross-sectional study of cultural and psychosocial aspects

**DOI:** 10.1016/j.jpra.2023.10.010

**Published:** 2023-10-20

**Authors:** Omar Fouda Neel, Hatan Mortada, Abdullah Q. AlAlwan, Reem Abdulmonem Al-Terkawi

**Affiliations:** a Division of Plastic Surgery, Department of Surgery, King Saud University, Riyadh, Saudi Arabia; b Division of Plastic Surgery, Department of Surgery, McGill University, Montreal, Canada; c Division of Plastic Surgery, Department of Surgery, King Saud University Medical City, King Saud University and Department of Plastic Surgery & Burn Unit, King Saud Medical City, Riyadh, Saudi Arabia; d College of Medicine, King Faisal University, Al Ahsa, Saudi Arabia; e Private Practice, Riyadh, Saudi Arabia

**Keywords:** Motivation, Social media, Aesthetic procedures, Aesthetic surgery, Female gender role stress, Life satisfaction

The growing quest for beauty has significantly influenced cosmetic surgery, which has seen a 1.5-fold increase worldwide in the past 20 years.^1^ The concept of beauty remains subjective, affected by various factors like culture and age.^2^ Yet, there is still no objective measure for facial attractiveness. Mass media perpetuates unrealistic beauty standards, negatively impacting body confidence and mental health. Almost half of all cosmetic surgery patients display mental health concerns, making psychological assessment critical for potential candidates.^3^ One key issue is Body Dysmorphic Disorder (BDD), where individuals have skewed perceptions of their body and often seek cosmetic procedures instead of psychiatric care.^4^ Understanding the psychological profile of patients is vital for their well-being. Our study explored the complex motivations behind choosing cosmetic surgery, such as the impact of societal expectations, self-esteem, and mental health conditions like BDD. We considered factors like gender role stress, life satisfaction, internalized beauty ideals, and opinions from loved ones. Our findings aim to offer a multi-faceted view of patient psychology, which can help tailor cosmetic surgery services to better meet patient needs and expectations.

This cross-sectional study investigated ten factors motivating cosmetic surgery, segmented into primary and secondary outcomes. Primary outcomes included female gender role stress and body dysmorphia, while secondary ones ranged from social media use to partner pressure. Patients attending post-op consultations at a private clinic in Riyadh were surveyed during November-December 2022. Informed consent was obtained, and the study received ethical clearance from the Institutional Review Board. Sociodemographic information such as age, marital status, and psychiatric history were collected. Primary outcomes were assessed using validated Likert scales. Body Dysmorphic Disorder was measured using the 7-item Dysmorphic Concern Questionnaire with scores ranging from 7 to 28. Female Gender Role Stress was assessed across five subdomains, with scores from 5 to 25. Secondary measures included health, life satisfaction, media influence, and social pressures, assessed via direct questions. RStudio was employed for analysis. Cronbach's alpha verified internal consistency for the primary outcomes. Descriptive statistics summarized the data, with additional tests for group comparisons and correlations between measures. The study used a robust methodology to achieve its aims, respecting both ethical considerations and statistical rigor, with outcomes measured through validated scales and direct questions.

Of 208 responses, 197 were included, with an 85.8 % female majority. Most participants were married (60.9 %), employed (61.9 %), and had a Bachelor's degree (65 %). Psychiatric disorders were reported by 10.7 %. 87.8 % had prior cosmetic procedures; 89.3 % were willing to undergo future ones. Median Female Gender Role Stress (FGRS) and Body Dysmorphic Disorder (BDD) scores were 10/25 and 12/28, respectively. Older age, having children, and residing in the central region increased willingness for cosmetic surgery; obesity decreased it. No association found with media influence, internalized beauty standards, or FGRS/BDD scores. FGRS positively correlated with BDD and negatively with self and life satisfaction. BDD also negatively correlated with these satisfaction metrics (Figure 1, Table 1). Life satisfaction was positively linked with self-health and negatively with media exposure.

This study examines motivations behind cosmetic surgery, focusing on factors such as gender, age, and psychological variables. Most participants were married, educated, employed women, consistent with global trends. Older and middle-aged women, particularly those with children, were more willing to undergo cosmetic procedures. Surprisingly, our study found no correlation between willingness for surgery and life satisfaction or media exposure. This contradicts prior research suggesting low self-esteem and high media exposure influence cosmetic surgery decisions.^5^ Moreover, our study didn't find Body Dysmorphic Disorder (BDD) and Female Gender Role Stress (FGRS) to significantly impact the willingness for cosmetic surgery, conflicting with other studies that noted these psychological factors as significant influencers. Social media appears to affect the perceived need for cosmetic procedures, but not necessarily the actual decision to undergo surgery. Future research should focus on ethical concerns and the role of mental health professionals in the decision-making process for aesthetic surgery.

Our study has limitations, including a small, non-diverse sample size and a lack of procedure-specific data, which may affect the generalizability and depth of findings. Nonetheless, it adds valuable insights into understanding patient motivations in Saudi Arabia and suggests directions for future studies, including the risks of cosmetic surgery and intervention measures.

In conclusion, this study found that older, married women are more inclined to undergo cosmetic surgery, while factors like media exposure and psychological stressors showed no clear correlation. The complexity of motivations calls for deeper, qualitative research to guide patient selection and counseling.

**Figure 1:**
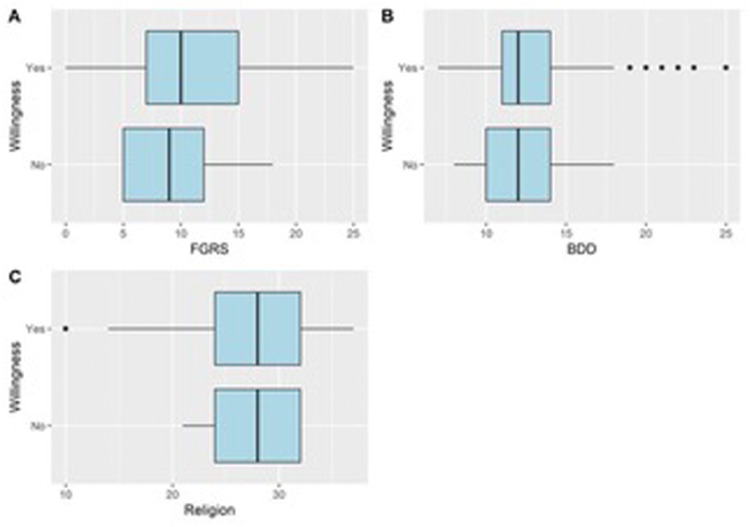
Boxplots showing the median scores of FGRS (A), and BDD (B) based on the status of participants’ willingness to undergo cosmetic surgeries.

**Table 1:** Factors associated with the willingness to undergo cosmetic surgeries.

Parameter	Category	Willingness		
		No, *N* = 21	Yes, *N* = 176	p-value
Age	Year	34.0 (28.0, 40.0)	40.0 (34.0, 47.0)	**0.027**
Gender	Male	6 (28.6 %)	22 (12.5 %)	0.089
	Female	15 (71.4 %)	154 (87.5 %)	
Relationship Status	Single	8 (38.1 %)	39 (22.2 %)	0.425
	Married	11 (52.4 %)	109 (61.9 %)	
	Divorced or separated	2 (9.5 %)	26 (14.8 %)	
	Widowed	0 (0.0 %)	2 (1.1 %)	
Educational level	Below high school	0 (0.0 %)	2 (1.1 %)	0.433
	High school	0 (0.0 %)	16 (9.1 %)	
	Bachelor's degree	14 (66.7 %)	114 (64.8 %)	
	Master's degree	4 (19.0 %)	33 (18.8 %)	
	Doctorate degree	3 (14.3 %)	11 (6.2 %)	
Occupation	Student	0 (0.0 %)	3 (1.7 %)	0.631
	Unemployed	3 (14.3 %)	34 (19.3 %)	
	Employee	16 (76.2 %)	106 (60.2 %)	
	Other	2 (9.5 %)	33 (18.8 %)	
BMI ≥ 30 kg/m^2^	Yes	6 (28.6 %)	21 (11.9 %)	**0.047**
Have children	Yes	8 (38.1 %)	119 (67.6 %)	**0.008**
City of residence	Central	14 (66.7 %)	152 (86.4 %)	**0.036**
	Western	1 (4.8 %)	9 (5.1 %)	
	Eastern	3 (14.3 %)	8 (4.5 %)	
	Northern	2 (9.5 %)	3 (1.7 %)	
	Southern	1 (4.8 %)	4 (2.3 %)	
Household Income	<5000 SR	3 (14.3 %)	16 (9.1 %)	0.831
	5000–10k SR	5 (23.8 %)	49 (27.8 %)	
	10k-20k SR	7 (33.3 %)	62 (35.2 %)	
	>20k SR	6 (28.6 %)	49 (27.8 %)	
Regular exercise	Yes	8 (38.1 %)	66 (37.5 %)	0.958
Ever diagnosed with a psychiatric disorder	Yes	2 (9.5 %)	19 (10.8 %)	>0.999

## Declaration of Competing Interest

The authors declare that they have no conflict of interest and have no commercial interest in the subject of study, nor receive any source of financial or material support.
